# Jdpd: an open java simulation kernel for molecular fragment dissipative particle dynamics

**DOI:** 10.1186/s13321-018-0278-7

**Published:** 2018-05-21

**Authors:** Karina van den Broek, Hubert Kuhn, Achim Zielesny

**Affiliations:** 1 Inorganic Chemistry and Center for Nanointegration, University of Duisburg-Essen, Essen, Germany; 2 CAM-D Technologies, Essen, Germany; 3 Institute for Bioinformatics and Chemoinformatics, Westphalian University of Applied Sciences, August-Schmidt-Ring 10, 45665 Recklinghausen, Germany

**Keywords:** Dissipative particle dynamics, Simulation, Molecular, Mesoscopic, Kernel

## Abstract

Jdpd is an open Java simulation kernel for Molecular Fragment Dissipative Particle Dynamics with parallelizable force calculation, efficient caching options and fast property calculations. It is characterized by an interface and factory-pattern driven design for simple code changes and may help to avoid problems of polyglot programming. Detailed input/output communication, parallelization and process control as well as internal logging capabilities for debugging purposes are supported. The new kernel may be utilized in different simulation environments ranging from flexible scripting solutions up to fully integrated “all-in-one” simulation systems.
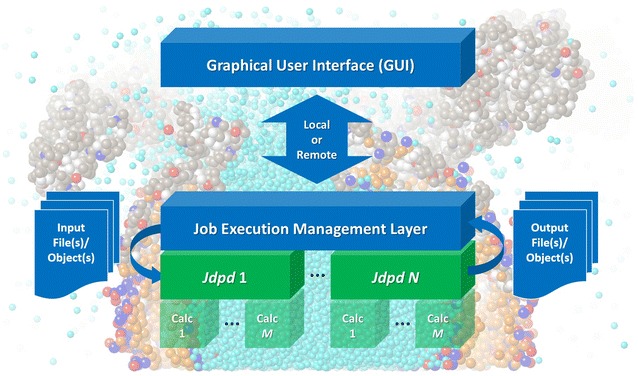

## Introduction

Mesoscopic simulation aims at describing supramolecular phenomena at the nanometer (length) and microsecond (time) scale for large interacting physical ensembles (representing millions of atoms) within comparatively short computational time frames (hours) by “coarse grained” neglect of uninteresting degrees of freedom. Dissipative Particle Dynamics (DPD) is a mesoscopic simulation technique for isothermal complex fluids and soft matter systems that combines features from Molecular Dynamics (MD), Langevin Dynamics and Lattice-Gas Automata [1–5]. It satisfies Galilean invariance and isotropy, conserves mass and momentum and achieves a rigorous sampling of the canonical ensemble due to soft particle pair potentials that diminish entanglements or caging effects. DPD is expected to show correct hydrodynamic behavior and to obey the Navier–Stokes equations.

Whereas DPD particles in general may be arbitrarily defined “fluid packets” [2] the Molecular Fragment DPD variant [5–12] identifies each particle with a distinct small molecule of molar mass in the order of 100 Da. Larger molecules are composed of adequate “molecular fragment” particles that are bonded by harmonic springs to mimic covalent connectivities and spatial 3D conformations. Thus this variant may be regarded as a chemically intuitive and molecular accurate “fine grained” version of the intrinsically “coarse grained” DPD technique.

A simulation kernel software comprises the fundamental data structures and numerical calculation algorithms that are necessary to approximate the temporal evolution of a defined particle ensemble. DPD (as well as similar MD) code consists basically of a main loop over (non-parallelizable) successive simulation steps in which (parallelizable) particle pair force evaluations are the most time-consuming part [13, 14]. Thus parallelization efforts focus mainly on these force calculations in order to speed up simulations.

**Fig. 1:**
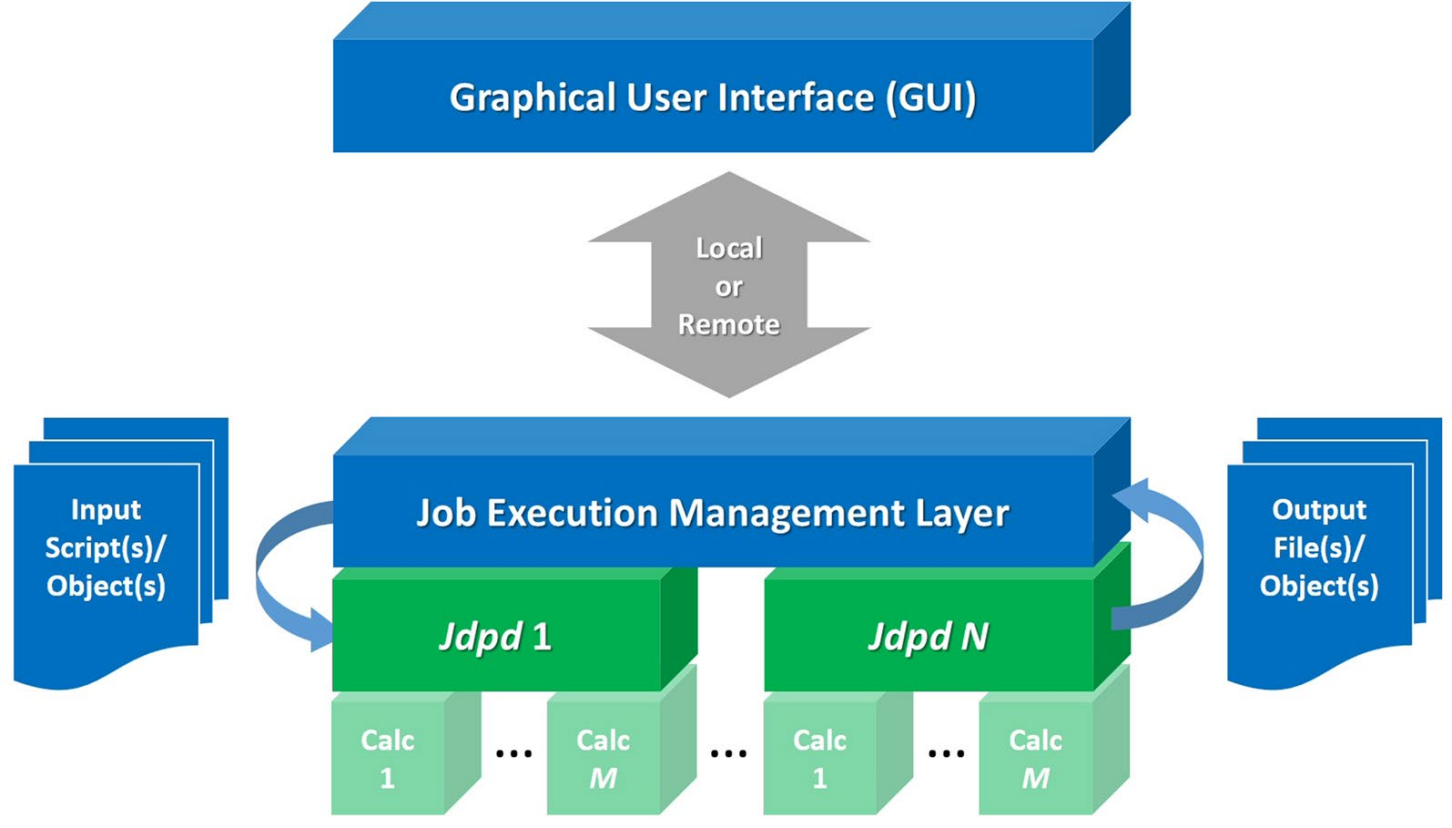
DPD simulation architecture(s) with Jdpd: A job execution management layer may run several parallelized Jdpd instances with different simulation jobs (denoted 1 to *N*) where every Jdpd instance itself may use several parallelized internal calculation threads (denoted Calc 1 to *M*)

The new Jdpd library enriches the small set of existing commercial [15, 16], open general [17–22] and hardware specific DPD kernels [23, 24]. It is (to our knowledge) the first pure Java implementation for Molecular Fragment DPD leveraging inherent Java strengths like cross-platform portability, parallelized computations, automatic memory management or object-oriented plus functional programming capabilities, see Fig. 1. In addition Jdpd may help to tackle problems of polyglot programming where a simulation kernel may be coded in C/C++ or FORTRAN with a managerial software layer written in a different language (like “universal” Java).

### Computational methods, implementation details and performance results

DPD particle trajectories are guided by Newton’s equation of motion [3, 5]:

$$ m_{i} \frac{{d^{2} \underline{r}_{i} }}{{dt^{2} }} = \underline{F}_{i} = \sum\limits_{\begin{subarray}{l} j = 1 \\ j \ne i \end{subarray} }^{N} {\left( {\underline{F}_{ij}^{C} + \underline{F}_{ij}^{D} + \underline{F}_{ij}^{R} } \right)} $$

$$ m_{i} ,\underline{r}_{i} , $$ mass and position vector of particle i; $$ t, $$ time; $$ \underline{F}_{i} , $$ total force on particle i exerted by other particles j; $$ N, $$ number of particles in simulation; $$ \underline{F}_{ij}^{C} ,\underline{F}_{ij}^{D} ,\underline{F}_{ij}^{R} , $$ conservative, dissipative and random force on particle i exerted by particle j.

Dissipative (frictional) and random forces oppose each other and act as a thermostat conserving the total momentum and introducing Brownian motion into the system. The conservative forces comprise soft DPD particle repulsions (with a common cut-off length of 1 DPD unit), harmonic springs between bonded particles and electrostatic interactions between charged particles (the implemented model for the latter is an ad-hoc approach to take electrostatic long-range interactions between “a few” charged particles in the simulation box into account—details are outlined in [12] where an application to biological membranes and protein models is described. A theoretically more sound treatment of electrostatic interactions like those proposed in [25–27] may be addressed by future developments with the current technical implementation of electrostatics interactions as a useful blueprint to alleviate coding efforts):

$$ \begin{aligned} \underline{F}_{ij}^{C} & = \underline{F}_{ij}^{C,DPD} + \underline{F}_{ij}^{C,Bond} + \underline{F}_{ij}^{C,Estat} \\ \underline{F}_{ij}^{C,DPD} \left( {\underline{r}_{ij} } \right) & = \left\{ {\begin{array}{*{20}l} {a_{ij} \left( {1 - r_{ij} } \right)\underline{r}_{ij}^{0} } & {{\text{ for }}r_{ij} < 1} \\ 0 & {{\text{ for }}r_{ij} \ge 1} \\ \end{array} } \right. \\ \underline{F}_{ij}^{C,Bond} & = - k_{Bond} \,\left( {r_{ij} - r_{Bond} } \right)\,\,\underline{r}_{ij}^{0} \\ \end{aligned} $$

$$ \underline{F}_{ij}^{C,DPD} ,\underline{F}_{ij}^{C,Bond} ,\underline{F}_{ij}^{C,Estat} , $$ soft repulsive DPD force, harmonic bond force and electrostatic force on particle i exerted by particle j; $$ a_{ij} , $$ maximum isotropic repulsion between particles i and j; $$ \underline{r}_{ij} = \underline{r}_{i} - \underline{r}_{j} = r_{ij} \,\underline{r}_{ij}^{0} $$; $$ \underline{r}_{ij}^{0} , $$ unit vector; $$ k_{Bond} , $$ spring constant of bond; $$ r_{Bond} , $$ bond length.

Dissipative force

$$ \underline{F}_{ij}^{D} \left( {\underline{r}_{ij} ,\underline{v}_{ij} } \right) = - \gamma \;\omega^{D} \left( {r_{ij} } \right)\;\left( {\underline{r}_{ij}^{0} \cdot \underline{v}_{ij} } \right)\;\underline{r}_{ij}^{0} $$

$\gamma ,$ friction coefficient; $\omega^{D} \left( {r_{ij} } \right),$ dissipative force distance variation; $\underline{v}_{i} ,$ velocity of particle i; $\underline{v}_{ij} = \underline{v}_{i} - \underline{v}_{j} .$

Random force

$$ \underline{F}_{ij}^{R} \left( {\underline{r}_{ij} } \right) = \sigma \;\omega^{R} \left( {r_{ij} } \right)\;\,\frac{{\zeta_{ij} }}{{\sqrt {\Delta t} }}\;\underline{r}_{ij}^{0} $$

$\sigma ,$ noise amplitude; $\omega^{R} \left( {r_{ij} } \right),$ random force distance variation; $\,\zeta_{ij} ,$ random number with zero mean and unit variance; $\Delta t,$ integration time step depend on each other in a canonical NVT ensemble (again a cut-off length of 1 DPD unit is applied) [5].

$$ \begin{aligned} \gamma &= \frac{{\sigma^{2} }}{{2\;k_{B} T}} \\ \omega^{R} \left( {r_{ij} } \right) &= \sqrt {\omega^{D} \left( {r_{ij} } \right)} = \left\{ {\begin{array}{*{20}c} {1 - r_{ij} } & {{\text{ for }}r_{ij} < 1} \\ 0 & {{\text{ for }}r_{ij} \ge 1} \\ \end{array} } \right. \\ \end{aligned} $$

$$ k_{B} , $$ Boltzmann constant; $$ T, $$ thermodynamic temperature.

The sketched forces and corresponding potentials are implemented in calculation classes of the packages *harmonicBonds, dpdCutoff1* and *electrostatics*. An additional gravitational acceleration that acts on particle masses may be defined for every direction. All calculation classes for DPD and electrostatics forces and potentials extend abstract class *ParticlePairInteractionCalculator* of package *interactions* which itself implements a cut-off length based simulation box cell partitioning [13]: The resulting cell linked-list method allows a (near) linear scaling detection of interacting particle pairs within neighbored cells—a task that otherwise would be quadratically scaling with the number of particles. In addition the simulation box cells are grouped in (3*3*2=)18 parallelizable chunks (each cell is surrounded by 26 neighbor cells, every third cell in every of the three spatial directions has no common neighbor cell but due to symmetric forces every second cell can be used in one of the three directions, see method *getParallelisationSafeCellChunks* of class *CellBox* in package *utilities*): These chunks guarantee a separated non-overlapping access of internal force array elements from parallelized calculation threads so that fast lock-free array manipulations become possible (all particle related arrays are located in class *ParticleArrays* of package *parameters*). An analogue lock-free parallelization feature with separated parallelizable bond chunks is realized within the bond related calculation classes (see package *harmonicBonds*).

Another significant performance improvement is achieved by efficiently caching the already evaluated interacting particle pairs for reuse throughout different force calculations within a single simulation step (see use of caching class *ParticlePairDistanceParameters* in package *utilities*). This caching avoids the time-consuming recalculation of particle pair distances (see relative performance factors of the different implemented integration schemata below): The saved number of expensively safe-guarded particle pair distance calculations may be (empirically) evaluated to be about 7 times the total number of particles in simulation for a common DPD particle density of 3. An upper bound for this empirical number may be deduced from the average number of all neighbor-cell particle pairs which is 40 times the number of particles in the simulation (which overestimates the number of relevant particle pair distances since—incorrectly—particle pairs with a distance above the cut-off length of 1 DPD unit are included):

$$\begin{aligned} \left\langle {N_{ij} } \right\rangle_{{\rho_{DPD} }} &= \underbrace {{\frac{N}{{\rho_{DPD} }}}}_{\substack{ {\text{Number of}} \\ {\text{cells}} } }\left( {\underbrace {{\frac{{\rho_{DPD}^{2} - \rho_{DPD} }}{2}}}_{\substack{ {\text{Number of particle pairs}} \\ {\text{within single cell}} } } + \underbrace {{13\rho_{DPD}^{2} }}_{\substack{ {\text{Particle pairs of single cell }} \\ {\text{with 13 of its 26 neighbor cells}} \\ ( {\text{no double counts)}} } }} \right) = \frac{{27\rho_{DPD} - 1}}{2}N \\ \\ \left\langle {N_{ij} } \right\rangle_{3} &= 40N \\ \end{aligned}$$

$$ N, $$ number of particles; $$ \rho_{DPD} , $$ DPD density; $$ \left\langle {N_{ij} } \right\rangle_{{\rho_{DPD} }} , $$ average number of particle pairs for specific DPD density.

Since (unlike MD) dissipative DPD forces depend on relative particle velocities the common Velocity-Verlet (VV) integration of the equations of motion [13, 14] has to be modified (abbreviated MVV). Jdpd consists of optimized implementations of four DPD integration schemata (located in package *integrationType*) that cover different integration techniques: (1) The original Groot-Warren scheme (GWMVV) [5, 28, 29] which depends on a tuning parameter where GWMVV equals VV integration for a value of 0.5, (2) the self-consistent scheme (SCMVV) [28–30] with an adjustable number of self-consistent dissipative force iterations where a single iteration leads to the DPDMVV variant, (3) Shardlow’s S1 scheme (S1MVV) [31, 32] and (4) the Nonsymmetric Pairwise Noose-Hoover-Langevin thermostat (PNHLN) [33] that requires the definition of an additional coupling parameter.

A single simulation task is performed by instantiating a *DpdSimulationTask* (which implements a Callable interface, see package *jdpd*) that may be submitted to an appropriate thread executor service to be invoked. The *DpdSimulationTask* constructor requires six configuration objects: (1) An (optional) *RestartInfo* instance that contains information about a possible simulation job restart (all Jdpd jobs may be restarted with altered settings which allows flexible job execution chains), (2) an input instance that implements the *IInput* interface with all simulation settings (e.g. particle types and their interactions, initial particle positions within the simulation box, additional properties like molecule boundaries etc.), (3) an output instance implementing the *IOutput* interface for all output data, (4) a progress monitor instance implementing the *IProgressMonitor* interface for real-time simulation progress information, (5) an *ILogger* instance for log-level dependent accumulation of detailed internal calculation progress information and (6) a *ParallelizationInfo* instance that describes internal settings for parallelized calculations. All interfaces are located in package *interfaces* and possess concrete sample implementations like classes *FileInput* and *FileOutput* of package *samples* that implement file-based I/O methods. The *RestartInfo* and *ParallelizationInfo* classes are found in package *parameters.*

An *IInput* object contains a *Factory* instance (located in package *utilities*) that implements enumerated type definitions for random number generation, DPD and electrostatics calculations, harmonic bonds or integration schemata. The factory pattern allows a simple extension or replacement of computational algorithms used throughout the whole simulation kernel, e.g. the implemented random numbers generators [34–36] may be supplemented by alternative methods with a few additional lines of code in the *Factory* class. An *IInput* object also provides detailed molecular information (see class *MoleculeDescription* in package *utilities*): These molecular descriptions differentiate between topological bonds of a molecules’ particles for an adequate description of covalent connectivity and bonds between backbone particles that maintain a defined spatial 3D structure, e.g. ring systems or secondary and tertiary structures of proteins. As a matter of course both bond types are treated equally throughout calculations.

For software programs with extensive parallelized numerical calculations the debugging facilities of current integrated development environments are often not sufficient to tackle subtle errors. Thus Jdpd comprises (extensible) loggers (see classes *FileLogger* and *MemoryLogger* in package *logger*) with different (extensible) log-levels to obtain detailed information about the simulation progress.

Jdpd efficiently calculates kinetic and potential energies, average kinetic temperatures [14], surface tensions along the simulation box axes [14, 37] or particle based radii of gyration [38]. In addition a fast parallelized particle and molecule based nearest-neighbor analysis for the whole simulation process is implemented which allows a detailed temporal monitoring of changing averaged particle’s and molecule’s vicinities. Initial force steps for potential energy minimization to improve start geometries may be defined and velocities may be scaled to keep a desired temperature. Molecule specific position or velocity fixation is provided with the additional option to define molecule specific boundaries in form of reflective virtual walls within the simulation box to confine molecules to a desired subspace. Molecule specific periodic “force kicks” may be defined to “smoothly drive” molecules into a desired direction. Periodic boundary conditions as well as reflective box walls are supported. Implemented safeguards try to tackle common problems like unfavorable simulation box start configurations with unphysical (high or low) particle densities.

For testing purposes Jdpd contains two appropriate packages *tests* and *tests.interactions* that contain Unit test code e.g. for a command file driven Jdpd usage.

**Fig. 2:**
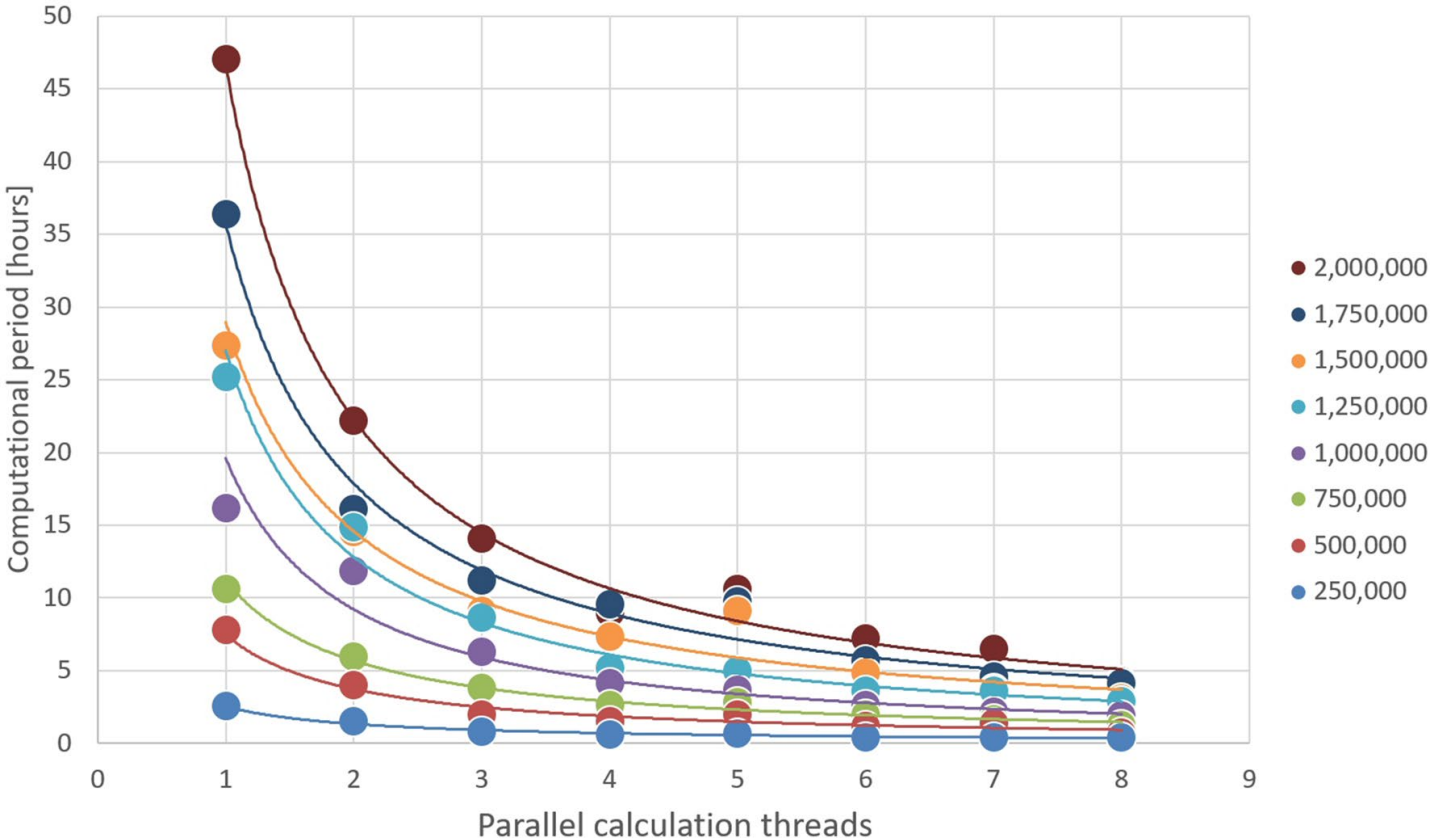
Jdpd performance snapshots in dependence of the number of simulated particles (see legend on the right) and the number of parallelized calculation threads for 13.000 simulation steps (corresponding to a physical time period of one microsecond) with integration type GWMVV

Jdpd offers a satisfactory performance for various scientific applications with workstation computers that are commonly available in scientific institutions (where the computational speed of Java just-in-time compiled Jdpd is in the ballpark of comparable ahead-of-time compiled FORTRAN or C/C++ code) but does not explicitly exploit specific hardware environments or devices like graphics processing units (thus Jdpd may be orders of magnitude slower than hardware specific high performance computing implementations—but future Java Virtual Machines may address these hardware acceleration options [39, 40]): As a rule of thumb a one-million-particle/one-microsecond simulation using 8 parallelized calculation threads consumes about one gigabyte of memory and takes less than 3 h to finish on standard multicore processors, e.g. an Intel Xeon E5-2697 v2 CPU [41] used for the performance snapshots shown in Fig. 2. Up to the studied 8 parallelized calculation threads Jdpd computational periods scale inversely proportional with the thread number exhibiting scaling exponents between − 0.92 and − 1.09. The corresponding scaling with a growing simulated particle number is near-proportional with scaling exponents between 1.17 and 1.39 (thus well below quadratic scaling). The relative performance factors of the different implemented integration schemata is GWMVV (1.0) < S1MVV (1.1) < DPDMVV (1.4) < S1MVV without cache (1.8) < PNHLN (1.9) < DPDMVV without cache (2.2) < SCMVV with 5 iterations (2.3) < PNHLN without cache (2.8) < SCMVV with 5 iterations without cache (4.8). These findings correspond to those reported in [28, 29, 32, 33].

## Conclusions

Jdpd is a new open DPD simulation kernel completely written in Java that complements the small available set of general purpose DPD kernels. It especially supports molecular fragment structures and offers parallelizable force calculation plus efficient caching options with an interface and factory-pattern driven design for comfortable and low expenditure code extensions, customizations or replacements. Detailed input/output communication, parallelization and process control as well as internal logging capabilities for debugging purposes are supported. The new kernel may be utilized in different simulation environments ranging from flexible scripting solutions up to fully integrated “all-in-one” simulation systems described in [11] where it may help to avoid polyglot programming.

The Jdpd library uses the Apache Commons RNG libraries [34] and is publically available as open source published under the GNU General Public License version 3 [42]. The Jdpd repository on GitHub comprises the Java bytecode libraries (including the Apache Commons RNG libraries), the Javadoc HTML documentation [43] and the Netbeans [44] source code packages including Unit tests.
